# Chemical and biological characterization of *Melaleuca subulata* (Cheel) Craven leaves’ volatile constituents supported by chemometric analysis and molecular docking

**DOI:** 10.1186/s12906-024-04345-0

**Published:** 2024-02-05

**Authors:** Heba E. Elsayed, Iriny M. Ayoub, Mohamed S. Mady, Fatma A. Moharram

**Affiliations:** 1 Department of Pharmacognosy, Faculty of Pharmacy, Helwan University, Cairo, 11795 Egypt; 2 Department of Pharmacognosy, Faculty of Pharmacy, Ain Shams University, Cairo, 11566 Egypt

**Keywords:** Antibacterial, Antioxidant, *Melaleuca sabulata*, Chemometrics, Essential oil, Molecular docking, Skin aging

## Abstract

**Background:**

The genus *Melaleuca* (Myrtaceae) comprises dozens of essential oil (EO)-rich species that are appreciated worldwide for their various medicinal values. Additionally, they are renowned in traditional medicine for their antimicrobial, antifungal, and other skin-related activities. The current study investigated the chemical profile and skin-related activities of volatile constituents derived from *M. subulata* (Cheel) Craven (Synonym *Callistemon subulatus*) leaves cultivated in Egypt for the first time.

**Methods:**

The volatile components were extracted using hydrodistillation (HD), headspace (HS), and supercritical fluid (SF). GC/MS and Kovat’s retention indices were implemented to identify the volatile compounds, while the variations among the components were assessed using Principal Component Analysis and Hierarchical Cluster Analysis. The radical scavenging activity was assessed using 2,2-diphenyl-1-picrylhydrazyl (DPPH), oxygen radical absorbance capacity (ORAC) and *β*-carotene assays. Moreover, the anti-aging effect was evaluated using anti-elastase, and anti-collagenase, while the antimicrobial potential was deduced from the agar diffusion and broth microdilution assays. Lastly, the molecular docking study was executed using C-docker protocol in Discovery Studio 4.5 to rationalize the binding affinity with targeted enzymes.

**Results:**

The SF extraction approach offered the highest EO yield, being 0.75%. According to the GC/MS analysis, monoterpene hydrocarbons were the most abundant volatile class in the HD oil sample (54.95%), with *α*-pinene being the most copious component (35.17%). On the contrary, the HS and SF volatile constituents were pioneered with oxygenated monoterpenes (72.01 and 36.41%) with eucalyptol and isopulegone being the most recognized components, representing 67.75 and 23.46%, respectively. The chemometric analysis showed segregate clustering of the three extraction methods with *α*-pinene, eucalyptol, and isopulegone serving as the main discriminating phytomarkers. Concerning the bioactivity context, both SF and HD-EOs exhibited antioxidant effects in terms of ORAC and *β*-carotene bleaching. The HD-EO displayed potent anti-tyrosinase activity, whereas the SF-EO exhibited significant anti-elastase properties. Moreover, SF-EO shows selective activity against gram-positive skin pathogens, especially *S. aureus*. Ultimately, molecular docking revealed binding scores for the volatile constituents; analogous to those of the docked reference drugs.

**Conclusions:**

*M. subulata* leaves constitute bioactive volatile components that may be indorsed as bioactive hits for managing skin aging and infection, though further in vivo studies are recommended.

**Supplementary Information:**

The online version contains supplementary material available at 10.1186/s12906-024-04345-0.

## Background

Essential oils (EOs) are fragrant, oily, hydrophobic liquids extracted from different parts of aromatic plants [1]. Owing to the plausible therapeutic applications of EOs, various conventional methods are known for their extraction such as hydrodistillation and solvent extraction [1]. Meanwhile, modern techniques have been continuously established to overcome the limitation of the conventional method, and to improve the extraction efficacy. Examples of the state-of-the-art approaches are the head space micro-extraction and super critical fluid extraction. The chemical profile of the extracted oil greatly varies concurrently with the applied extraction technique. For this reason, the selection of the optimal method depends on the required oil quality and composition correlated to the proposed therapeutic value [2]. For instance, head space analysis offers a potentially express method that requires symbolic plant material [3]. Conventionally applied approaches, as steam distillation and solvent extraction, result in excessive losses of volatile constituents [4]. Yet, super critical liquid extraction offers reliable oil with minimal degradation byproducts and efficient recovery. In terms of EOs' medicinal value, the antioxidant and antibacterial properties of volatile constituents have been acknowledged. An advantage that won't ever be noticeably reduced with time. After the revolution in the “golden era”, when almost crucial antibiotics were discovered, history repeats itself nowadays, and the current antimicrobial agents are in danger of losing their effectiveness due to increasing microbial resistance [5]. Shortly, failures in the treatment associated with multidrug-resistant bacteria have become a global issue for public health [6]. For this reason, the search for new antibiotics is a vital objective to control the clinical threat and decrease the associated morbidity and mortality. Interestingly, plants can provide an enormous range of complex and structurally diverse natural products. Hence, several scientists have focused on the study of their extracts, essential oils, and secondary metabolites as potential antimicrobial agents [7]. The antimicrobial fundamentals of essential oils (EOs) are well documented which almost depends on the nature of EO, the functional pharmacophores in its chemical constituents, and the targeted organisms [8]. Owing to their hydrophobic nature, EOs disturb the coherence structure of the cell wall and cytoplasmic membrane, making them more permeable. The membrane permeability leads to the outflow of cellular materials followed by cell death. Moreover, EOs can damage lipids and proteins by coagulating the cytoplasm [9].

Free radicals and other reactive oxygen species cause hazardous oxidation of biomolecules resulting in a phenomenon called oxidative stress [10]. Cellular components are subjected to damage and misbalance, which eventually leads to molecular malfunction associated with various chronic disorders such as aging [10]. The free radical concept suggests that age-related damage at the cellular level occurs through various mechanisms like membrane lipid peroxidation, formation of age-related pigments, and cross-linkage of proteins [11]. Hence, interventions intended for free radicals’ regulation or inhibition should be able to decrease the rate of aging with subsequent reduction in disease pathogenesis. Antioxidants, particularly naturally-derived ones, can accomplish this mission expertly owing to the multifunctional pharmacophores that are employed in various compartments. Naturally derived antioxidants master radical scavengers by counteracting the free radicals, reducing the peroxide concentrations, repairing the oxidized membranes, and inhibiting lipid metabolism [12]. Among the commonly known natural products with remarkable antioxidant activity/free radical scavenging, are essential oils (EOs). As per epidemiological studies, which show a massive increase in free radical-related malfunctions, hence the discovery and identification of new essential oil-based antioxidants is in eminent demand.

Genus *Melaleuca*, belonging to the Myrtle family (Myrtaceae), comprises mainly 290 species of aromatic shrubs and small trees native to Australia [13]. The native Australian communities traditionally use these species as antiseptic agents [14], while their EOs are known as food flavors [15]. On the other side, *Melaleuca* species are recognized in folk medicine for their medicinal value in the management of cough, treatment of bronchitis, and as a remedy for skin and gastrointestinal tract infections [14]. Regarding the previously reported biological activities, *Callistemon’s* EOs are induced with many medicinal values such as antimicrobial, antithrombosis, larvicidal effects, and anti-inflammatory [14, 15]. *Melaleuca sabulata* (Cheel) Craven (commonly known as bottlebrush) is a small shrub native to Australia and cultivated in Egypt for its ornamental value. However, there is little information about its phytochemical and biological value except for the antibacterial and anticancer activities of its essential oil as well as the polyphenolic profile of its leaves [16–18].

In continuation of our research on *M. subulata* (Cheel) Craven (synonym *Callistemon subulatus*) cultivated in Egypt, we report for the first time the comparative GC/MS chemical profile coupled to chemometrics of the essential oils extracted from the leaves using three different approaches *viz* hydrodistillation, headspace, and supercritical fluid extraction. Furthermore, the antibacterial, antioxidant, antiaging, and whitening potential of the hydrodistilled and supercritical extracted EOs were determined and correlated to the identified volatiles. Besides, in silico molecular docking was conducted to unravel the possible binding interactions of the identified volatiles to the targeted enzymes.

## Materials and methods

### General

All chemicals, reagents, and Nunc Micro-well™ plates were purchased from Sigma Aldrich (Milan, Italy and St. louis, MO, United states) except otherwise stated. Oxygen radical absorbance capacity was recorded using FLUOstar OPTIMA (Franka Ganske, BMG LABTECH, Offenburg, Germany), while ELX 808microplate reader (BioTek, Italy) was adopted to measure the absorbance’s in the other assays. The extracted volatile constituents by hydro distillation and supercritical fluid were analyzed on Shimadzu GC/MS-QP2010 linked to a quadrupole mass spectrometer (Shimadzu Corporation, Kyoto, Japan) supplemented with Rtx-5MS column (30 m × 0.25 mm i.d. × 0.25-μm film thickness, Restek, United States). For the antimicrobial assays, the stock cultures of Gram-positive bacteria, *Staphylococcus aureus* (ATCC 25923)*, Streptococcus pyogenes* (ATCC 12344), *Clostridium perfringens* (ATCC 13124), and the Gram-negative *Pseudomonas aeruginosa* (ATCC 9027) were supplied from Microlab, Institute of Research and Technology (Vellore, Tamilnadu, India). Mueller–Hinton agar and broth, biological grade sterile DMSO, chloramphenicol (C), and gentamycin (CN) 6.0 mm discs (positive control antibiotic) were purchased from Oxoid, Thermo Fisher Scientific (MA, United States).

### Plant material

The leaves of *M. subulata* (Cheel) Craven were harvested at the flowering stage of an ornamental perennial tree at Orman Botanic Garden, Giza, Egypt (March 2021) after the endorsement of the local garden`s guidelines, and the collection rules of Egypt. Morphological authentication of the plant was done by Dr. Trease Labib, Consultant of Plant Taxonomy at Mazhar Botanical Garden, Giza, Egypt. A voucher sample was approved after authorities’ permission and deposited at the Herbarium of the Pharmacognosy Department, Faculty of Pharmacy, Helwan University, Cairo, Egypt under deposition number 05 Msu/2021. All the applied experiments and methods on the investigated plant comply with the institutional, national, and international guidelines and legislation.

### Preparation, and identification of volatile constituents

#### Hydrodistillation

*M. subulata* fresh leaves (750 g) were subjected to hydrodistillation extraction as previously stated [19]. Briefly, the leaves were grounded, mixed with distilled water, and extracted for 4 h using a Clevenger apparatus, and the process was repeated till exhaustion. The separated EO was dried over anhydrous Na_2_SO_4_ and then analyzed using gas chromatography-mass spectrometry (GC/MS) following the conditions set formerly [19] and briefly described in supplementary data.

#### Supercritical fluid

EOs extraction using CO_2_ gas as supercritical fluid (SF) was implemented following the reported procedure by our research team [19]. Briefly, 400 g dried leaves were extracted using supercritical CO_2_ and ethanol (as co-solvent) at 40 °C and 15.0 MPa for 1 h in a static mode followed by 1 h in dynamic mode. The obtained extract was dried over anhydrous Na_2_SO_4_ then analyzed using GC/MS following the conditions set formerly [20] and briefly described in supplementary data.

#### Dynamic head-space

The detection of volatile constituents from the fresh leaves sample using dynamic head-space (HS) was carried out as per the standard procedure stated in the literature [21]. About 2 g of *M. subulata* leaves were placed into a 5 mL glass vial of a Shimadzu headspace sampler HS-20 coupled to a Shimadzu GCMS-QP2020 gas chromatograph mass spectrometer (Koyoto, Japan) equipped with Rtx-1MS column (30 m × 0.25 mm id. × 0.25 µm film thickness) (Restek, Bellefonte, PA, USA). The analysis was accomplished following the conditions set formerly [21] and briefly described in the supplementary data.

#### Identification of the volatile components

Each oil sample was analyzed individually in triplicate and the mean value of the data was recorded. Identification of the essential oil components was tentatively determined on the basis of their retention indices (RI) relative to standard *n*-alkanes (C_8_-C_28_), and matching their mass spectra with that in the NIST Mass Spectral Library (2017) and Wiley Registry of Mass Spectral Data 8th edition, in addition to comparison with previously reported data (similarity index > 90%) [22–25].

### Multivariate data analysis

Analysis of unsupervised multivariate data was achieved using the Unscrambler X10.4, CAMO software (Computer Aided Modeling, AS, Norway). Principle component analysis (PCA) and hierarchical cluster analysis (HCA) were applied to give insights into the relative phytochemical variability amongst *M. subulata* volatile constituents extracted by different approaches. Cluster analysis was conducted by Ward's method. The distances between clusters were assessed using the squared Euclidean method [26].

### Antioxidant capacity

#### 2-Diphenyl-1-picrylhydrazyl radical scavenging assay

The DPPH radical scavenging capacity of the HD and SF extracted EOs was assessed by following the protocol described previously by our research team [27]. In short, 100 µL of different concentrations of the oil samples or the positive control (ascorbic acid) were added to an equivalent amount of DPPH solution. The mixtures were mixed for 30 min and the absorbances were measured at λ_517_ on a microplate reader.

#### Oxygen radical absorbance capacity

The oxygen radical absorbance capacity (ORAC) of the extracted EOs was carried out in accordance with the procedure described earlier [27]. Briefly, different concentrations of oil samples were added to 10 mM phosphate buffered (pH 7.4) and a fluorescein dye. The time taken till the decay of the fluorescence from each sample was measured as it is equivalent to its ORAC, compared to Trolox as a positive control.

#### *β*-Carotene bleaching assay

Inhibition of lipid peroxidation was determined as per the method delineated in the literature using Butylhydroxytoluene (BHT) as a reference standard drug [27]. Different concentrations of the tested EOs, were added to a mixture of *β*-carotene, linoleic acid, and Tween 20, and the absorbances were measured on a microplate reader at λ_max_ 470 nm according to the manufacturer’s protocol.

### Anti-aging and whitening activity

#### Anti-elastase assay

The ability of tested EOs to inhibit the activity of elastase enzyme was evaluated following the protocol described by Ebrahim and Co-workers [27]. Concisely, in a 96-well plate, different concentrations of the oil samples or an elastase reference inhibitor were incubated in HEPES buffer with 1 μg/mL elastase enzyme at 25 °C. Twenty minutes later, 1 mM MeO-SucAAPVpNA (100 μL) was added as substrate and the absorbances were measured at λ_405_ nm on a microplate reader.

#### Anti-collagenase assay

The ability of the tested EOs to inhibit the activity of the collagenase enzyme was accomplished according to the method mentioned previously by Ebrahim et al. [27]. Collagenase enzyme (1 mg/mL in 50 mM tricine buffer) was incubated at 37 °C with different concentrations of the tested oil samples or EDTA (as collagenase inhibitor) for twenty minutes. Thereafter, 100 μL FALGPA (an amino acid substrate) was added to each tested sample and incubated at 37 °C for one hour followed by the addition of 200 μL 2% ninhydrin and 200 μL isopropanol. Ultimately, the absorbance was measured at λ_540_ nm using a microplate reader.

#### Anti-tyrosinase assay

The anti-tyrosinase potential of the tested EOs (tested conc. 25–300 µL/mL) in comparison to kojic acid as tyrosinase inhibitor standard was estimated as per the procedure described by Ebrahim et al. [27]. Simply, different concentrations of the EO samples were incubated with tyrosinase enzyme and 1 mM L-DOPA (as a substrate) for 15 min at 37 °C. The absorbance of each sample was measured on a microplate reader at λ_475_ nm.

### Antibacterial activity against skin-related pathogenic bacteria

#### Agar-well diffusion assay

The susceptibility of selected skin-related pathogens to the tested EOs was carried out using the standard agar well-diffusion method following the Clinical and Laboratory Standards Institute protocol [28]. About 10 × 10^4^ cells of each reference strain were cultured on Muller Hinton agar plates. Thereafter, 0.6 cm diameter wells were formed using a sterile cork-borer. 50 µL of different concentrations of each EO sample were added to each well and incubated for twenty-four hours. The diameter of the developed inhibition zones was measured in mm and compared to reference antibiotics as positive controls and DMSO as a negative control.

#### Determination of minimum inhibitory concentration

The broth microdilution assay was implemented to measure the minimum inhibitory concentration (MIC) of the tested EOs against the previously mentioned bacterial strains. EOs were prepared as 100 µL/mL DMSO stock solutions after which they were diluted to 1/10 in sterile Mueller Hinton broth. The experiment was accomplished based on the protocol described earlier [29].

### In silico molecular docking study

In silico molecular docking was performed employing C-docker protocol in Discovery Studio 4.5 (Accelrys Inc., San Diego, CA, USA). The X-ray crystal structures of three potential target enzymes involved in the ageing process viz collagenase (PDB ID: 465C; 2.40 Å), human neutrophil elastase (PDB ID: 1H1B; 2.00 Å), and tyrosinase (PDB ID: 5M8Q; 2.85 Å) were retrieved from the protein data bank [30]. Enzymes were prepared following the default protocol in Discovery Studio [31–33]. In brief, water molecules were removed except for those involved in the binding to the inhibitor and hydrogen atoms were added. The protein structure was refined. CHARMm force field was adopted and MMFF94 was chosen for partial charge calculation with subsequent energy minimization of the target protein. The co-crystallized ligands were used to define the active binding sites in the target enzymes. Ligands were removed prior to docking simulations. Volatile compounds annotated in *M. subulata* were retrieved from Pubchem [34] and subsequently prepared by ligand preparation protocol in Discovery Studio. The prepared ligands were docked into the active sites of the energy-minimized protein using C-Docker protocol. Besides, EDTA was docked in collagenase, N-(methoxysuccinyl)-Ala-Ala-Pro-Val-chloromethyl ketone in elastase, whereas kojic acid was docked in tyrosinase enzyme. Free binding energies were calculated in kcal/mol as previously stated by Ayoub et al. [30]. Validation of C-Docker protocol was achieved by re-docking each co-crystalized inhibitor into the active site of its enzyme, followed by calculation of the root-mean-square deviations (RMSDs) between the co-crystalized ligand and its docked pose.

### Statistical analysis

All data were done in triplicates and averaged from three independent experiments. Values were expressed as mean ± SD and the IC_50_ of each tested sample was calculated from the non-linear regression analysis from the curves plotted between log sample concentration and the measured absorbance or fluorescence implemented on GraphPad Prism version 5.0 (San Diego, CA, United States).

## Results and discussion

Essential oils (EOs) are considered as a complex mixture of volatile metabolites that are recognized by their distinctive chemical scaffold, unique aroma, and valuable applications [35]. Despite their rich and complex structure, the use of EOs remains paramount and inclusive to the cosmetics and perfumery fields, and to a lesser extent to aromatherapy, however their therapeutic value and health benefits still need further exploitation. In general, each plant yields its “signature” of EOs components, which differ according to the plant organ and its geographical locality. Such variation could affect the biological activity of the oil in either synergistic, additive, or antagonistic manner [36]. Also, the selection of the appropriate method to extract the EOs depends on several factors. For instance, hydrodistillation (HD) represents the most common and cheap method, but the composition of the resulting oil can be affected by several issues such as isomerization, saponification, and or polymerization [37]. On the other side supercritical fluid (SF) is a green technology that generates high-quality EO in a considerable yield [38]. Accordingly, herein we report the extraction of the volatile constituents of *M. subulata* leaves, cultivated in Egypt, using the HD, SF, and HS methods for the first time, to compare their analysis results in terms of chemical and biological aspects. The results revealed that different preparation methods affect not only the color of the oil but also its yield. For instance, the HD EO is dark yellow with a highly pleasant, mint-like odor, while possessing dark brown, faint pleasant, highly viscous extract in SF. Meanwhile, the highest yield was observed by the SF extraction (0.75%) being three-folds more than the HD yield (0.26%), while the HS oil was unrecoverable. In all, the yield, and organoleptic properties of the obtained EOs were almost consistent with the reported pros and cons of each technique. In particular, the supercritical CO_2_ used in SF extraction is non-viscous, possesses low surface tension, endorses high diffusion power, and significant yield [39]. Moreover, HD multilateral extraction process, is useful for large or small industries in which prolonged distillation produces only a small amount of essential oil. Ultimately, the HS technique addressed only the issue of direct quantifying volatiles in challenging solid matrices but with a net result of negative yield [40].

Additionally, the extraction techniques were likewise affecting the composition of *M. subulata* leaves in many aspects. For instance, a total of 19 (97.50%) and 16 (99.58%) volatile components were recognized in HD and HS derived EO and aroma, respectively while 31 compounds constituting 47.12% were known in the SF-derived extract (Table 1, Supplementary Figs. S1, S2, S3). Moreover, the variability in the class and percentage of the identified volatiles was noticeable; in the case of HD EO, *α*-pinene (35.30%) and eucalyptol (1,8-cineol, 34.42%) represent the main components, also both compounds represented the major ones in case of HS but with different percentage being 67.75% and 15.46% for 1,8 cineol and *α*-pinene, respectively. In the case of SF extraction method, the identified volatile compounds were totally different from the HD and HS, since isopulegone represented the major compound (23.46%) followed by squalene (6.81%). Concerning the calculated percentage of each chemical class, it is interesting to notice that there is a considerable variation, as oxygenated compounds percentage being 42.21, 71.83, and 34.49 for HD, HS, and SF, respectively, while that of non-oxygenated components was 55.29 (HD), 27.75 (HS), and 12.63 (SF). In addition, the monoterpene hydrocarbons (MH) and sesquiterpene hydrocarbons (SH) percentage were variable among the adopted methods, with the highest percentage of MH being observed in HD-EO (55.16%). While the lowest is in SF-EO (4.54%). Moreover, the HS volatile components encompassed the major percentage of oxygenated monoterpene (OM), being 70.24% in comparison to HD (39.56%) and SF (29.21%). Our HD results were in accordance with the previously published data [16, 17], however they varied in the estimated quantitative percentage of the identified volatile components which may be, at least in part, due to seasonal and geographical variations [41]. Moreover, the explanation for the high percentage of OM in the volatile constituents extracted using state-of-the art approaches, just as the HS and SF, may be attributed to the minimized extraction time, low heating temperature, and absence of water which in all reduces the level of degradation of oxygenated compounds and preserve their proportion [17].

**Table 1:** Average percent concentration (%) of the volatile components in *Melaleuca subulata* (Cheel) Craven leaves extracted using hydrodistillation (HD), headspace (HS), and supercritical fluid (SF) extraction methods

No.	Compound	MF	M. wt.	RI_Exp_^[a]^	RI_Lit_^[b]^	HD	HS	SF
						Avergare % Conc. (n=3)		
1.	2-Hexenal	C_6_H_14_O	98	827	832	**------**	1.13	**------**
2.	Isoamyl acetate	C_7_H_14_O_2_	130	859	860	**------**	0.12	**------**
3.	Isobutyl isobutyrate	C_8_H_16_O_2_	144	898	895	0.35	0.40	**------**
4.	*α*-Thujene	C_10_H_16_	136	909	909	1.85	0.80	1.86
5.	*α*-Pinene	C_10_H_16_	136	918	918	35.17	15.46	0.24
6.	Sabinene	C_10_H_16_	136	966	966	**------**	1.21	**------**
7.	*β*-Pinene	C_10_H_16_	136	980	980	1.36	**------**	**------**
8.	*β*-Myrcene	C_10_H_16_	136	987	987	1.20	1.50	**------**
9.	*α*-Phellandrene	C_10_H_16_	136	994	994	6.81	2.78	2.73
10.	3-Carene	C_10_H_16_	136	1000	1000	0.20	**------**	**------**
11.	(+)-4-Carene	C_10_H_16_	136	1007	1004	0.24	**------**	0.11
12.	*p*-Cymene	C_10_H_14_	134	1015	1015	5.92	1.49	0.23
13.	*o-*Cymene	C_10_H_14_	134	1015	1015	**------**	**------**	0.13
14.	Eucalyptol	C_10_H_18_O	154	1022	1022	34.32	67.75	1.89
15.	Limonene	C_10_H_16_	136	1025	1025	**------**	4.15	0.13
16.	*trans*-*β*-Ocimene	C_10_H_16_	136	1033	1033	**------**	0.06	**------**
17.	*cis*-*β-*Ocimene	C_10_H_16_	136	1039	1039	0.34	0.23	**------**
18.	γ-Terpinene	C_10_H_16_	136	1049	1049	1.17	0.42	**------**
19.	*α*-Terpinolene	C_10_H_16_	136	1079	1063	0.69	**------**	**------**
20.	Linalool	C_10_H_18_O	154	1090	1090	**------**	0.23	0.06
21.	Pinocarveol	C_10_H_16_O	152	1130	1130	0.24	**------**	**------**
22.	Terpinen-4-ol	C_10_H_18_O	154	1168	1168	4.87	2.38	0.12
23.	*α-*Terpineol	C_10_H_18_O	154	1183	1183	**------**	**------**	2.67
24.	5-Hydroxy-2,2,6,6-tetra-methyl-4-cyclohexene-1,3-dione	C_13_H_18_O_4_	238	1256	1464.4	**------**	**------**	1.27
25.	Caryophyllene	C_15_H_24_	204	1410	1410	0.26	**------**	**------**
26.	Aromadendrene	C_15_H_24_	204	1431	1431	**------**	**------**	0.62
27.	*Allo-*aromadandrene	C_15_H_24_	204	1454	1454	**------**	**------**	0.22
28.	*β-*Humulene	C_15_H_24_	204	1489	1456	**------**	**------**	0.57
29.	*α-*Bisabolol	C_15_H_26_O	284	1503	1681	**------**	**------**	0.10
30.	Cinerolon	C_10_H_14_O_2_	166	1513	1641	**------**	**------**	0.45
31.	Epiglobulol	C_15_H_26_O	222	1553	1580	0.15	**------**	0.34
32.	Isopulegone	C_10_H_16_O	152	1544	1173	**------**	**------**	23.46
33.	(-)-Spathulenol	C_15_H_24_O	220	1571	1572	0.32	**------**	0.91
34.	Globulol	C_15_H_26_O	222	1577	1577	1.64	**------**	0.65
35.	4-Caranone	C_10_H_16_O	152	1636	1197	**------**	**------**	0.59
36.	*α*-Phellandrene-dimer	C_20_H_32_	272	1784	1801.4	**------**	**------**	0.66
37.	Dehydrocostuslactone	C_15_H_18_O_2_	230	1991	2006.7	**------**	**------**	0.39
38.	Phytol	C_20_H_40_O	296	2099	2103	**------**	**------**	1.34
39.	Behenic alcohol	C_22_H_46_O	326	2271	2456	**------**	**------**	0.21
40.	2-Methylhexacosane	C_27_H_56_	380	2575	2656	**------**	**------**	0.06
41.	Squalene	C_30_H_50_	410	2801	2814	**------**	**------**	6.44
42.	*γ-*Sitosterol	C_29_H_50_O	414	3030	3290	**------**	**------**	0.42
43.	*dl*-*α*-Tocopherol	C_29_H_50_O_2_	430	3081	3112	**------**	**------**	0.37
44.	*β-*Sitosterol	C_29_H_50_O	414	3279	3203	**------**	**------**	0.59
45.	Phytyl decanoate	C_30_H_58_O_2_	450	3517	2956.5	**------**	**------**	0.58
	**Total identified compounds **					97.10	100	50.41
	**Non-oxygenated compounds**					55.21	28.10	14.00
	Monoterpene hydrocarbons (MH)					54.95	28.10	5.43
	Sesquiterpene hydrocarbons (SH)					0.26	------	1.41
	Others					------	------	7.16
	**Oxygenated compounds**					41.89	72.01	36.41
	Oxygenated monoterpenes (OM)					39.43	70.36	29.24
	Oxygenated sesquiterpenes (OS)					2.11	------	2.39
	Others					0.35	1.65	4.78

*MF* Molecular formula, *M. wt.* Molecular weight, *RIExp* experimental retention index, *RILit* reference retention index

**Fig. 1:**
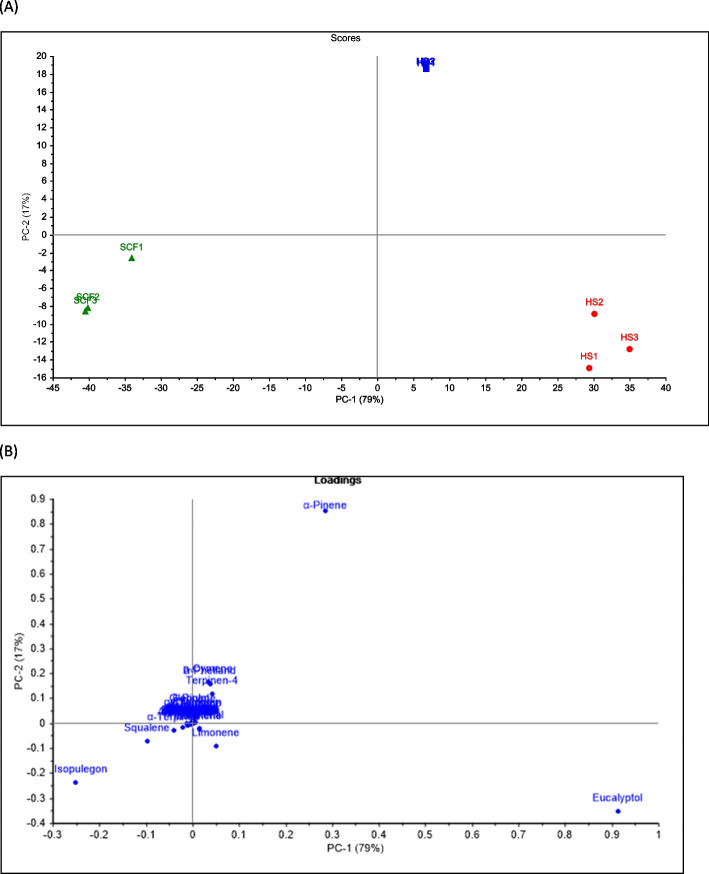
Principal component analysis of the phytochemical profile of *M. subulata* hydrodistilled essential oils (HD), headspace aroma (HS) and supercritical fluid extracts (SF): (**A**) score plot and (**B**) loading plot with the contributing metabolites assigned

GC/MS-based data acquired for the volatiles identified from HD, HS, and SF extractions were combined in PCA score plot. Two orthogonal PCs were established which collectively explained 96% of the total variance among the samples where PC1 accounted for 79% of the variance and PC2 for 17% (Fig. 1A). EOs prepared by HD were separately clustered in the upper right side of the score plot with positive PC1 values. Besides, HS samples were clustered in the lower right side of the score plot, being separated by the negative side of PC2. Meanwhile, volatiles prepared by SF extraction were clustered separately in the lower left quadrant in the negative side of PC1 and PC2. The loading plot (Fig. 1B) depicts the metabolites responsible for the segregation observed herein where *α*-pinene, eucalyptol, and isopulegone were the major discriminating phytomarkers. *α*-Pinene was the main metabolite responsible for the segregation of the HD samples, positively contributing to PC1 and PC2. Besides, eucalyptol was abundant in accessions sampled by HS having a negative contribution to PC2. On the other hand, the segregation of SF extract could be ascribed to isopulegone, which was absent in volatile components obtained by HD or via HS and contributed negatively to PC1 and PC2.

**Fig. 2:**
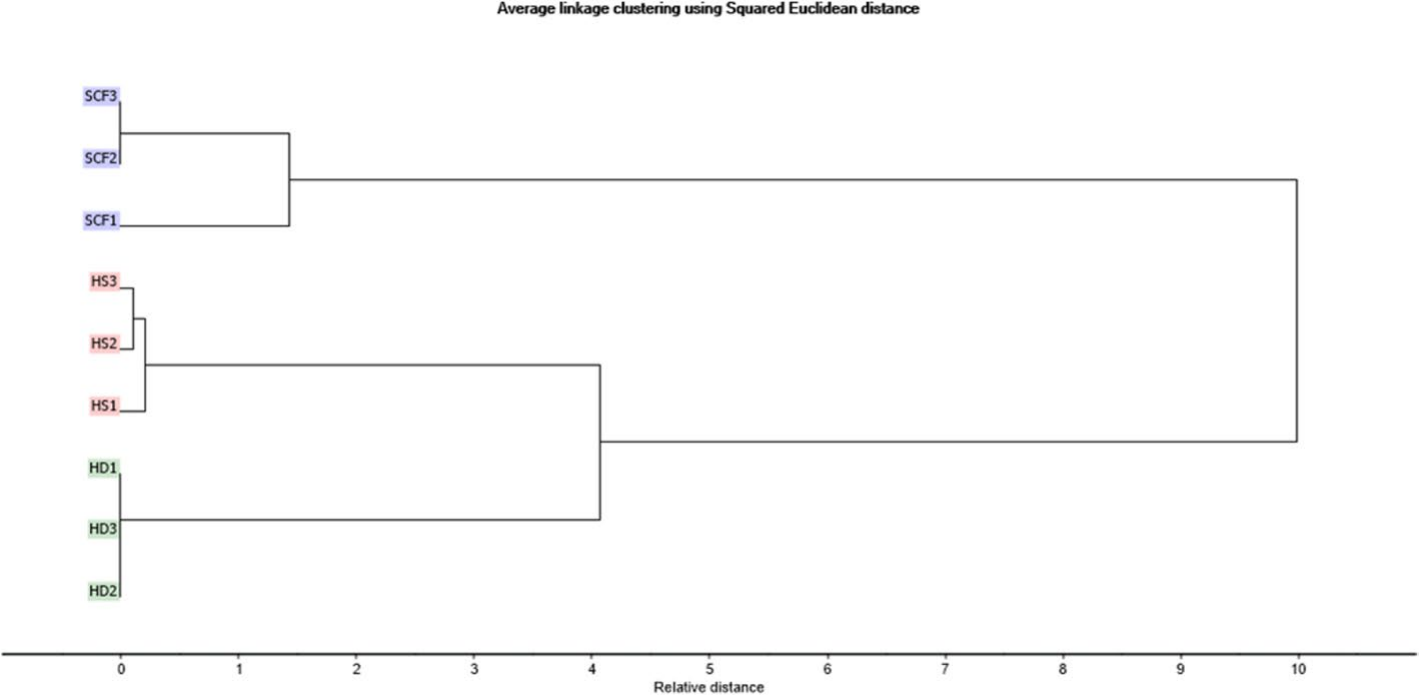
Hierarchical cluster analysis (HCA)-derived dendrogram of *M. subulata* hydrodistilled essential oils (HD), headspace aroma (HS) and supercritical fluid extracts (SF) based on the phytochemical profile analyzed by GC/MS

Besides, HCA clustering was performed to further confirm the results obtained from PCA, in which samples were clustered into two main clusters, as shown in HCA dendrogram (Fig. 2). Both HS and HD-volatile constituents were clustered together (cluster II) with a short distance between them, compared to the volatiles obtained by SF extraction, which formed a separate cluster (cluster I). Hence, the HCA dendrogram ascertained the results demonstrated by PCA, revealing that the volatiles composition obtained by HS and HD are more closely correlated chemically to each other.

The aging process can be provoked by endogenous or exogenous agents and is largely associated with oxidative stress, through the formation of reactive oxygen species (ROS) [10, 11]. ROS directly impairs skin cells, mediates inflammatory responses, and contributes to degradation of essential extracellular matrix components. Hence, topical antioxidant application can be quite beneficial since it prevents molecular damage and maintains skin homeostasis. In this regard, testing the antioxidant potential of *M. subulata* EOs is a valuable strategy. Various in vitro tests are implemented to evaluate the antioxidant activity of natural products, though they differ in their sensitivity and specificity. So, the application of different analytical methods is ideal to evaluate the effectiveness and the antioxidant mechanism of potential hits [42, 43]. Accordingly, the co-application of three methods, categorized as enzyme-based assays, to evaluate the antioxidant activity of *M. subulata* leaves’ EOs was suggested. DPPH radical scavenging, and oxygen radical absorbance capacity (ORAC), both rely on electron transfer machinery, while the *β*-carotene bleaching depends on hydrogen atom transfer [44]. Our findings (Table 2) showed that the tested extracts possessed weak DPPH radical scavenging capacity with an IC_50_ 18.5 ± 2.45 and 15.0 ± 0.43 µL/mL for HD and SF samples, respectively, in comparison to ascorbic acid (IC_50_ 1.83 µg/mL). On the other side, the mean value of ORAC for the tested oil samples showed significant potent activity with IC_50_ 17.0 ± 3.34 µL/mL (HD) and 16.0 ± 0.38 µL/mL (SF) which are supreme to that of the trolox (27.0 ± 13.41 µg/mL). Lastly, the results of the *β*-carotene bleaching assay showed that the HD (IC_50_ = 8.41 ± 0.67 µL/mL) has comparable inhibitory activity as BHT (IC_50_ 8.06 ± 0.67 µg/mL) while the SF-derived oil (5.28 ± 0.69 µL/mL) is more potent than BHT. Our results highlighted the promising antioxidant capacity of the tested samples, although the DPPH radical scavenging assay was inequitable in unveiling the findings. This could be due to the low miscibility of EO’s in the assay reagents so, the DPPH could not be suitable for the evaluation of the antioxidant activities of the EO’s [45]. In fact, the HD EO contains major constituents such as eucalyptol (1,8-cineole) and *α*-pinene which have been reported to display antioxidant activities, at least in part, due to their unique chemical scaffold [46]. *α*-Pinene is a monoterpene hydrocarbon possessing strongly activated methylene groups which are probably responsible for its antioxidant potential [47]. Concurrently, 1,8-cineole is related to oxygenated monoterpenes which are well known for their antioxidant activity [48]. On the other side, the antioxidant activity of the SF–EO may be attributed to its isopulegone content which encompasses promoting structural features as the exomethylene of the vinyl group as well as the neighborhood of an activated *α*-hydrogen to the ketone carbonyl. Since EOs often consist of a complex mixture of volatile components, it is possible for minor compounds to have a substantial role in the oil activity through a synergistic mechanism with the major components [49]. That may also rationale the convergent activity of the HD and SF EO’s, though they differ qualitatively and quantitively, in their major components. For instance, *p*-cymene, an aromatic monoterpene identified in both samples, is renowned for its promising antioxidant effect. p-Cymene’s antioxidant mechanism summarized in scavenging of reactive species such as hydroxyl radical and nitrite oxide, hence prevents the oxidation of biomolecules [50]. Also, Barra and co-workers reported that EO rich in terpinen-4-ol, *p*-cymene, and *α*-pinene possessed promising antioxidant properties [51]. Lastly, the antioxidant capacity of *α*-phellandrene (a cyclic monoterpene) has been documented in the ferric reducing/antioxidant power (FRAP), and the nitric oxide scavenging activity (NO^•^) [52]. In conclusion, the antioxidant capacity of a tested sample depends on the applied protocol, the physicochemical properties, and the combined contribution of the constituted components [48–53].

**Table 2:** Antioxidant capacity of the extracted EOs from *Melaleuca. subulata* (Cheel) Craven leaves against reference standard drugs in DPPH, ORAC, and *β*-carotene in vitro assays

Tested sample	IC_50_ ± SD (µL/mL)		
	DPPH	ORAC	*β-C*arotene
HD	18.5 ± 2.45	17.0 ± 3.34	8.41 ± 0.67
SF	15.0 ± 0.43	16.0 ± 0.38	5.28 ± 0.69
Ascorbic acid^a^	1.83 ± 1.41	-	-
Trolox^a^	-	27.0 ± 13.41	-
BHT^a^	-	*-*	8.06 ± 0.67

^a^µg/mL

Tyrosinase is a well-distributed enzyme in human tissue which plays a vital role in melanin production. Mutations in melanogenesis have assumedly been connected with skin hyperpigmentation and cancer [54]. Consequently, inhibition of tyrosinase could probably contribute to clinical therapies for skin cancers and other dermatological syndromes. Meanwhile, the exposure of the skin to external harmful factors such as UV radiation and temperature resulting in the increase of the enzymes complicated in the aging process, such as collagenase and elastase. They trigger the degradation of main components such as collagen and elastin. This in turn speeds up the skin visible aging proved by age-related skin changes as wrinkles and sagging skin [55]. Herein, the extracted EOs were investigated for them in vitro antiaging and whitening capacity in relation to their inhibitory effect on elastase, collagenase, and tyrosinase enzymes, which are strongly correlated to the diminishing of elasticity and integrity of the epidermal tissues. Our finding (Table 3) revealed that the HD-EO exhibited significant anti-tyrosinase activity with IC_50_ 290.19 ± 2.59 µg/mL in comparison to Kojic acid (321.65 ± 3.41 µg/mL) which in accordance with previously published data that the EO with the relatively low-oxygenated terpenoids displayed better tyrosinase inhibitory activity [56]. On the contrary, the SF derived EO showed better anti-elastase activity (IC_50_ 54.18 ± 1.12 µg/mL), than the HD-EO (IC_50_ 63.13 ± 1.62 µg/mL), in comparison to the standard elastase inhibitor drug (IC_50_ 44.92 ± 1.71 µg/mL). Ultimately, a similar profile was observed for the tested EOs which showed moderate anti-collagenase with IC_50_ 392.07 ± 1.75 µg/mL (HD) and 362.26 ± 2.84 µg/mL (SF) in comparison to EDTA as reference standard drug (IC_50_ 315.12 ± 2.21 µg/mL, Table 3). Several reports highlighted that collagenase and elastase inhibition may result from the suppression of pro-inflammatory mediators in addition to the antioxidant potential of the applied treatments. In the same context, our results mirrored significantly, promising anti-aging activities which are almost correlated to the antioxidant potential of its volatile components and correlated to their ability to protect the different skin layers [55].

**Table 3:** Anti-aging IC_50_ (%inhibition) of the extracted EOs from *Melaleuca subulata* (Cheel) Craven leaves against reference standard drugs in anti-elastase, anti-collagenase, and anti-tyrosinase enzyme-based assays

Tested samples	IC_50_ ± SD (% inhibition ± SD)		
	Anti-elastase	Anti-collagenase	Anti-tyrosinase
HD	63.13 ± 1.62 (80.15% ± 1.28)	392.07 ± 1.75 (53.12% ± 2.46)	290.19 ± 2.59 (85.89% ± 1.60)
SF	54.18 ± 1.12 (85.12% ± 1.32)	362.26 ± 2.84 (62.35% ± 2.1)	353.5 ± 1.03 (62.03% ± 1.85)
MeOSu-AAPV-CMK^a^	44.92 ± 1.71 (92.52% ± 4.63)	-	-
EDTA^a^	-	315.12 ± 2.21 (79.82% ± 2.63)	-
Kojic acid^a^	-	-	321.65 ± 3.41 (76.52% ± 0.83)

^a^µg/mL

Given the opportunity of searching for new antimicrobial agents from natural sources as they are often considered as safe in comparison to industrial chemicals, EOs are notable as being promising antimicrobial leads. In our study, we tested the effect of HD and SF EOs against dermatological gram positive-pathogens including *S. aureus* which produce a wide variety of clinical manifestations including bacteremia, skin, and soft tissue infections, *S. pyogenes*, which is an aerotolerant bacteria, usually cause Group A streptococcal skin infection as well as, *Clostridium perfringens* which causes tissue necrosis, bacteremia, emphysematous cholecystitis, and gas gangrene [57–59]. In addition to *P. aeruginosa* (Gram-negative pathogen) which is most frequently associated with an opportunistic infection, varies from skin-localized infections to life-threatening systemic disease [60]. Infections usually occurred both in community or hospital-acquired locations and the treatment remains challenging to achieve due to the emergence of multi-drug resistant strains [57]. The results showed that the Gram-positive strains are more susceptible to the tested EOs in a dose response dependent-manner (Table 4, Supplementary Figs. S4 and S5), while the Gram-negative bacteria being resistant to the applied treatments. Generally, the observed differential activity is due to the presence of a peptidoglycan layer which lies outside the bacterial outer membrane. Whereas the outer membrane in gram-negative bacteria, is composed of a double layer of phospholipids linked with lipopolysaccharides inner membrane, thus hydrophobic macromolecules as EO’s constituents, become unable to penetrate the double membrane and Gram-negative bacteria developed instant resistance [61]. Interestingly, the SF derived EO sample attained larger inhibitory zones (18–31 mm) than the HD oil (7–20 mm), which even exceeds the inhibitory zone of the standard reference antibiotic chloramphenicol (9–18 mm) at the maximum tested dose (20 µL/mL, Table 4). Meanwhile, the SF-EO displayed potent MIC being 2.5 µL/mL for *S. aureus* and 5.0 µL/mL for* S. pyogenes* and *C. perfringens*, respectively (Table 5). Else way, the HD-EO possessed MIC (MIC = 5–10 µL/mL, Table 5) which is almost two-fold less active than the SF (MIC = 2.5–5 µL/mL, Table 5). The potent activity of the SF oil sample is almost correlated to its privileged, oxygenated monoterpenoids ketone represented by isopulegone which reportedly its antimicrobial activity in the skimmed literature [62, 63]. On the other hand, the HD-EO displays lower antimicrobial activity than the SF sample due to its high percentage of *α-*pinene (a monoterpene hydrocarbon) which was previously known that it possesses low antimicrobial activity. This data was in accordance with previous studies that documented the low antimicrobial potential of hydrocarbons in general. Meanwhile, oil constitutes a moderate percentage of eucalyptol, which belongs to oxygenated terpenoids, that are famous for having more intense antimicrobial activity than other constituents [64]. Hence, synergistic, or antagonistic effects between some components, in all, may affect the antimicrobial activity of the tested samples.

**Table 4:** Zones of inhibition of the extracted EOs from *Melaleuca subulata* (Cheel) Craven leaves against reference skin-related microbial pathogens in comparison to standard antibiotics using agar well diffusion assay

Reference strains	Tested EOs/ Conc. µL/mL						Tested antibiotics/ Conc. µg/mL			
	**HD**			**SF**			**C**	**CN**	**CEC**	**FOX**
	**5**	**10**	**20**	**5**	**10**	**20**	**30**	**10**	**30**	**30**
**Gram positive Bacteria**										
*S. aureus (ATCC 25923)*	12 mm	17 mm	20 mm	18 mm	20 mm	28 mm	13 mm	^−^	^−^	^−^
*S. pyogenes (ATCC 12344)*	7 mm	8 mm	12 mm	19 mm	24 mm	29 mm	9 mm	10 mm	^−^	^−^
*C. perfringens (ATCC 13124)*	Nz	12 mm	18 mm	24 mm	28 mm	31 mm	18 mm	12 mm	^−^	^−^
**Gram negative Bacteria**										
*P. aeruginosa (ATCC 9027)*	Nz	Nz	Nz	Nz	Nz	Nz	9 mm	^−^	^−^	^−^
10% DMSO (-ve control)	Nz	Nz	Nz	Nz	Nz	Nz	^−^	^−^	^−^	^−^

*C* Chloramphenicol, *CN* Gentamycin, *CEC* Ceftriaxone, *FOX* Cefoxitin, *Nz* No zone of inhibition was observed

^−-^not tested

**Table 5:** Minimum inhibitory concentrations (MICs) of the extracted EOs from *Melaleuca subulata* (Cheel) Craven leaves against reference skin-related Gram-positive pathogens using microdilution broth assay

Susceptible organisms	MIC (µL/mL)	
	**HD**	**SF**
*S. aureus (ATCC 25923)*	5	2.5
*S. pyogenes (ATCC 12344)*	10	5
*C. perfringens (ATCC 13124)*	10	5

To unravel the possible binding mechanism of the identified compounds to the target enzymes collagenase, elastase, and tyrosinase, in silico molecular docking studies were conducted. The results (Table 6) showed that a variety of compounds exhibited strong docking scores with the studied proteins.

**Table 6:** Free binding energies (∆G) of the volatile components identified in the EOs of M*. subulata* leaves extracted using hydro-distillation (HD), headspace (HS), and supercritical fluid (SF) within the active sites of collagenase, elastase and tyrosinase enzymes using molecular docking and expressed in kcal/mol

No	Compound	C-docker energy ∆G (Kcal/mol)		
		**Collagenase**	**Elastase**	**Tyrosinase**
1	2-Methyl hexacosane (**40**)	-61.35	- 37.26	f.d
2	N-(Methoxysuccinyl)-Ala-Ala-Pro-Val-chloromethyl ketone	-	- 41.59	-
3	Behenic alcohol (**39**)	- 58.30	- 39.52	f.d
4	EDTA	- 51.65	-	-
5	dl-*α*-Tocopherol (**43**)	- 39.98	- 26.34	f.d
6	Phytol decanoate (**45**)	- 34.54	- 20.13	f.d
7	Isobutyl isobutyrate (**3**)	- 27.06	- 22.29	-26.78
8	Isoamyl acetate (**2**)	- 23.19	- 23.93	-23.15
9	Phytol (**38**)	**-** 22.31	- 12.62	-9.39
10	*p*-Cymene (**12**)	- 21.91	- 16.74	-21.52
11	*o*-Cymene (**13**)	- 19.79	- 13.74	-20.67
12	2-Hexenal (**1**)	- 15.77	- 9.30	-19.77
13	4-Caranone (**35**)	- 11.19	- 2.86	-8.85
14	Isopulegone (**32**)	- 4.43	- 2.04	-3.98
15	Pinocarveol (**21**)	- 0.46	3.29	-2.21
16	*β*-Pinene (**7**)	1.10	6.56	-0.83
17	*α*-Terpineol (**23**)	4.46	11.08	7.94
18	α-Pinene (**5**)	5.27	9.34	3.78
19	Eucalyptol (**14**)	6.15	10.43	5.83
20	Linalool (**20**)	6.21	11.34	3.88
21	4-Carene (**11**)	7.87	14.29	8.75
22	*α*-Phellandrene (**9**)	8.25	11.75	10.95
23	Terpinen-4-ol (**22**)	9.16	11.45	9.88
24	*β*-Myrcene (**8**)	12.47	13.92	12.97
25	3-Carene (**10**)	13.34	17.71	13.97
26	Epiglobulol (**31**)	14.26	16.18	13.27
27	cis-*β*-Ocimene (**17**)	15.12	18.75	21.55
28	Trans-*β*-ocimene (**16**)	15.53	19.87	18.55
29	Caryophyllene (**25**)	15.75	17.43	15.16
30	Globulol (**34**)	15.80	22.04	13.59
31	*α*-Bisabolol (**29**)	18.10	29.82	30.06
32	Cinerolon (**30**)	18.85	26.01	22.89
33	Limonene (**15**)	19.66	22.10	22.69
34	Allo-aromadandrene (**26**)	24.83	29.69	19.55
35	*γ*-Terpinene (**18**)	24.98	28.39	24.55
36	Aromadendrene (**26**)	27.58	31.43	25.47
37	*α*-Thujene (**4**)	31.24	35.72	31.10
38	*α*-Terpinolene (**19**)	33.56	39.86	37.21
39	*β-*Humulene (**28**)	37.13	41.18	42.03
40	*γ*-Sitosterol (**42**)	43.03	41.71	f.d
41	Spathulenol (**33**)	43.39	38.42	19.47
42	*β*-Sitosterol (**44**)	44.56	41.75	f.d
43	Dehydrocostuslactone (**37**)	46.62	49.77	44.13
44	*α*-Phellandrene dimer (**36**)	81.06	83.46	78.94
45	Squalene (**41**)	88.45	98.74	f.d
46	Diphenyl-ether sulphone based hydroxamic acid (456C inhibitor)	- 52.69	-	-
47	Gw475151 (1H1B inhibitor)	-	- 7.34	-
48	Kojic acid (5M8Q inhibitor)	-	-	-16.79

*f.d.* failed to dock; Positive values indicate unfavorable interaction

**Fig. 3:**
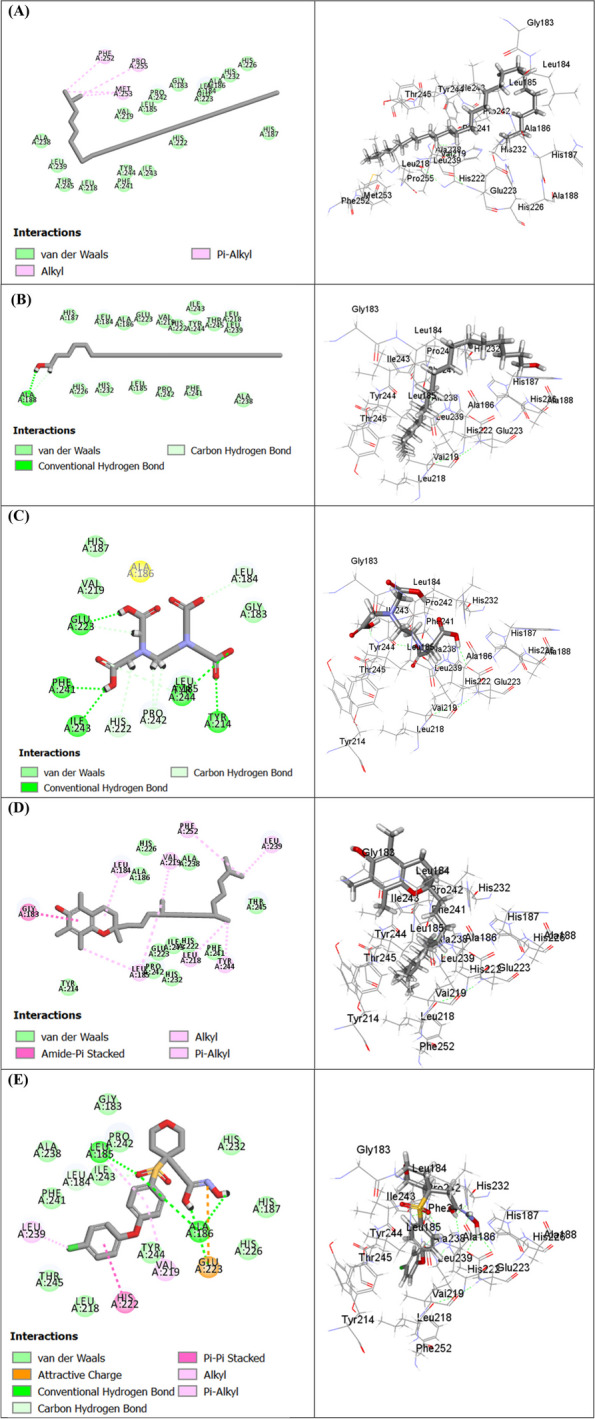
2D and 3D binding modes of 2-methyl hexacosane (A), behenic alcohol (B), EDTA (C), dl-*α*-Tocopherol (D) and 465C co-crystallized inhibitor (E) within the active sites of collagenase enzyme

For instance, 2-Methyl hexacosane (**40**), behenic alcohol (**39**) (Fig. 3) in the SF EO showed favorable binding within the active sites of collagenase enzyme (456C) with free binding energies of -61.35 kcal/mol and -58.30 kcal/mol, respectively, displaying higher docking scores than diphenyl-ether sulphone-based hydroxamic acid, the co-crystallized inhibitor (-52.69 kcal/mol) and EDTA, the standard drug used in the in vitro assay (-51.65 kcal/mol). dl-*α*-tocopherol (**43**), phytol decanoate (**45**) and the major compound isopulegone (**32**) in SF extract also showed favorable binding exhibiting free binding energies of -39.98 kcal/mol, -34.54 kcal/mol, and -4.43 kcal/mol, respectively. This could explain the observed in vitro collagenase inhibitory activity of SF derived EO.

**Fig. 4:**
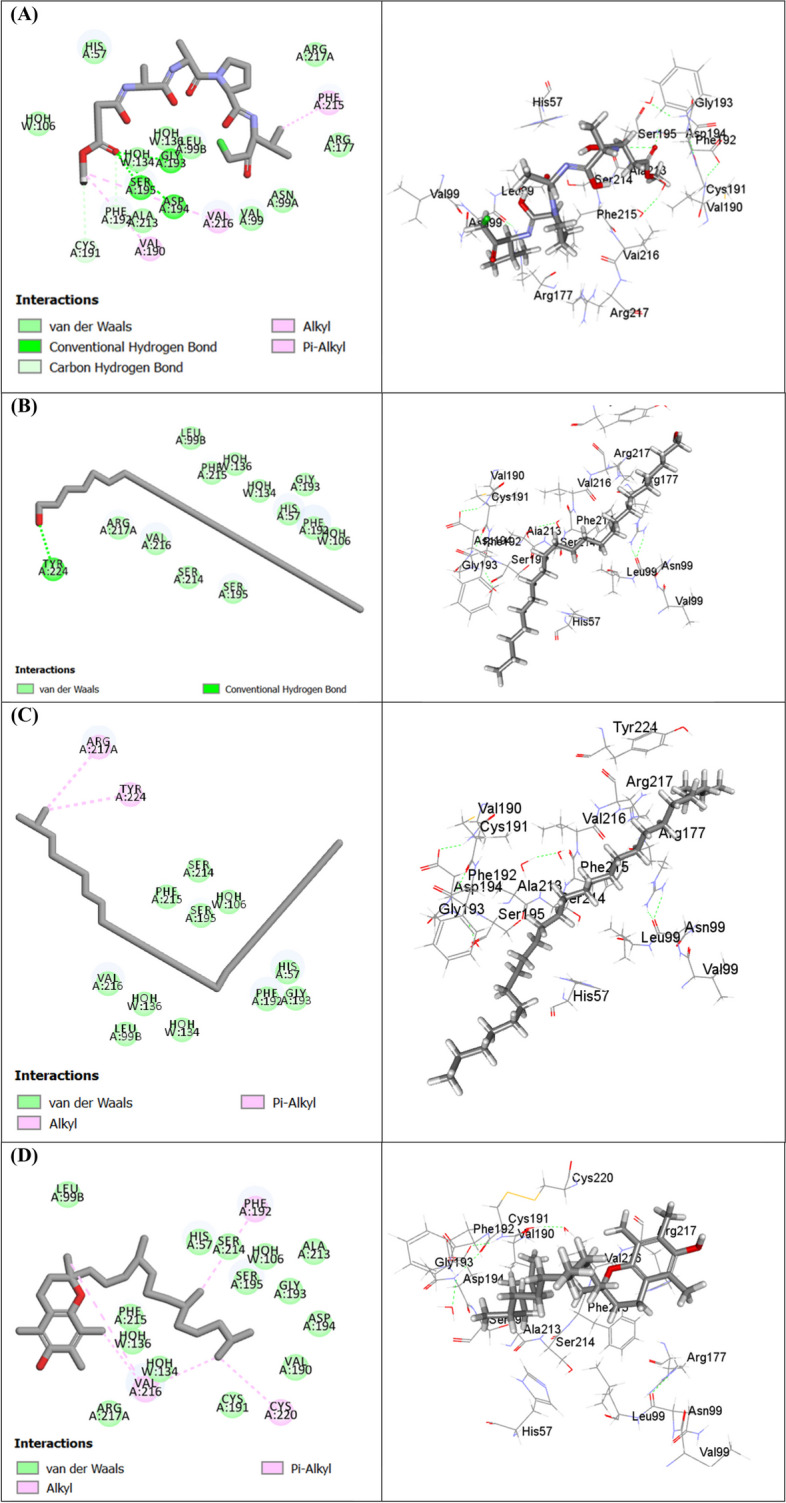
2D and 3D binding modes of N-(methoxysuccinyl)-Ala-Ala-Pro-Val-chloromethyl ketone (**A**) behenic alcohol (**B**) 2-methyl hexacosane (**C**) dl-α-tocopherol (**D**) within the active sites of elastase enzyme

In the same context, compounds identified in SF extract showed better anti-elastase activity where 2-methyl hexacosane (**40**), behenic alcohol (**39**), *dl*-*α*-tocopherol (**43**), phytol decanoate (**45**), and isopulegone (**32**) displayed favorable binding to the active sites of elastase enzyme (Fig. 4) exhibiting free binding energies of -39.52 kcal/mol, -37.26 kcal/mol, -26.34 kcal/mol, -20.13 kcal/mol and -2.04 kcal/mol, respectively. Besides, the co-crystallized inhibitor displayed a free binding energy of -41.59 kcal/mol.

**Fig. 5:**
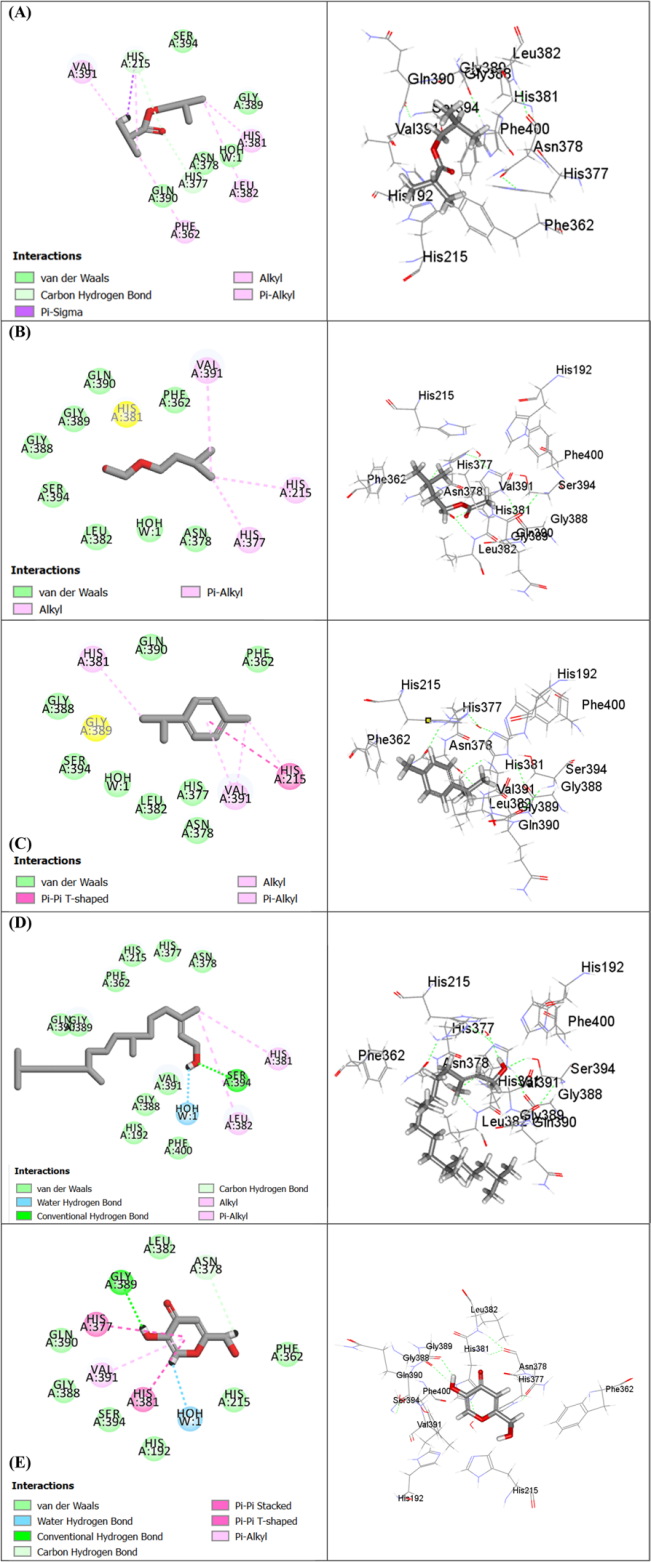
2D and 3D binding modes of isobutyl isobutyrate (**A**) isoamyl acetate (**B**) *p*-cymene (**C**) phytol (**D**) and kojic acid (**E**) within the active sites of tyrosinase enzyme

On the other hand, examination of in silico tyrosinase inhibitory activity, the compounds identified in HD EO (Fig. 5) displayed best scores when compared to those in SF extract with isobutyl isobutyrate (-26.78 kcal/mol) and *p*-cymene (-21.52 kcal/mol) showing better scores than kojic acid, the co-crystallized inhibitor, and the reference standard (-16.79 kcal/mol) indicating favorable binding to tyrosinase enzyme active site. The high fitting scores exhibited by *M. subulata* identified compounds could be endorsed mainly to the formation of Van der Waals interactions with amino acid residues at the active sites of the enzymes, in addition to occasional conventional H-bonds, C-H bonds, alkyl and π-alkyl interactions.

An interesting observation from the molecular docking findings is that a panel of minor volatile components have shown favorable binding affinities (docking scores) with the investigated proteins (enzymes). Due to their extended confirmation and bulkiness that enabled their full occupation and interaction to both proximate and even distant amino acids within the binding pocket of the targeted protein [65]. Yet highlighted the non-negligible or perhaps the substantial synergistic role of these components with the major volatiles in the observed in vitro bioactivities. On the other side, some major volatile components such as eucalyptol and *α*-pinene showed unfavorable binding affinities. A remark that coincides with previous report by Altyar et al. [66] who documented the free binding energies (∆G) for *α*-pinene as 5.27 and 9.34 kcal/mol in the active sites of collagenase and elastase enzymes, respectively. The unfavorable binding may be attributed to lacking some protein–ligand interactions including ionic, hydrogen bonds, and van der Waals interactions. Another reason that may be considered is the limitation of docking a flexible ligand to a rigid receptor conformation, which sometimes provides results that may not correlate with the experimental in vitro simulation [67]. In all, though molecular docking was acknowledged to unveil the binding affinity of every single compound, the complex nature of EO which is the base of their multi-target activity should not be ignored.

## Conclusion

The chemical composition of volatile constituents extracted from *M. subulata* leaves cultivated in Egypt, showed compositional variations mutual to the applied extraction approaches. The chemo diversity among the different extracts was further evaluated using unsupervised multivariate data analysis, where *α*-pinene, eucalyptol and isopulegone represented the major discriminating phytomarkers contributing to their segregation. The synergism between the whole volatile components, rather than single purified one, may be correlated to the selective antimicrobial, antioxidant, skin whitening, and anti-skin aging capacity of *M. subulata* leaves EOs. In silico molecular docking study was carried out to report the binding affinity and energy of the recognized compounds with the target enzymes In all, *M. subulata* leaves EO may be promoted as bioactive hit for the management of dermatological disorders related to aging and infection, however further ex vivo studies are required.

### Supplementary Information

**Additional file 1.**

## Data Availability

All data generated or analyzed during this study are included inside the manuscript and/or its supplementary information files.

## Abbreviations

BHT: Butylhydroxytoluene
DPPH: 2,2-Diphenyl-1-picrylhydrazyl radical
EO: Essential oil
GC/MS: Gas chromatography/Mass spectrometry
HCA: Hierarchical cluster analysis
HD: Hydrodistillation
HS: Head-space
MIC: Minimum inhibitory concentration
ORAC: Oxygen radical absorbance capacity
PCA: Principal component analysis
ROS: Reactive oxygen species
SF: Supercritical fluid
ZOI: Zone of inhibition
