# Supplementary material for: Chemical and biological characterization of Melaleuca subulata (Cheel) Craven leaves’ volatile constituents supported by chemometric analysis and molecular docking
Source: BMC Complement Med Ther. 2024 Feb 5;24:76. doi: 10.1186/s12906-024-04345-0 (PMC10840179; doi:10.1186/s12906-024-04345-0)
Supplement: Supplementary file 1 — Additional file 1. [file 12906_2024_4345_MOESM1_ESM.docx]

**Supplementary data**

**
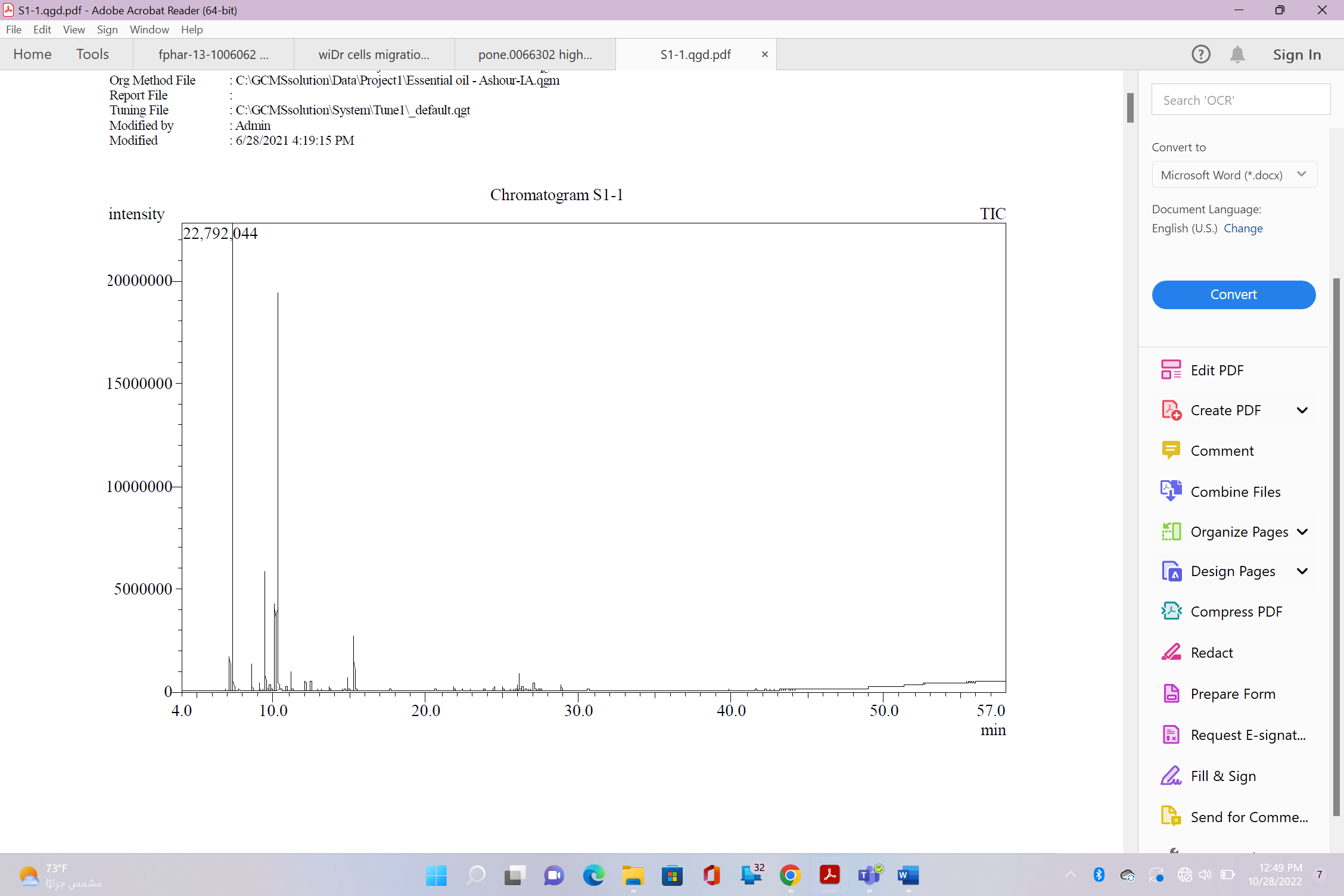
**

**Figure S1 A: Total ion chromatogram for *M. subulata* essential oil obtained by hydrodistillation (HD) extraction**

**
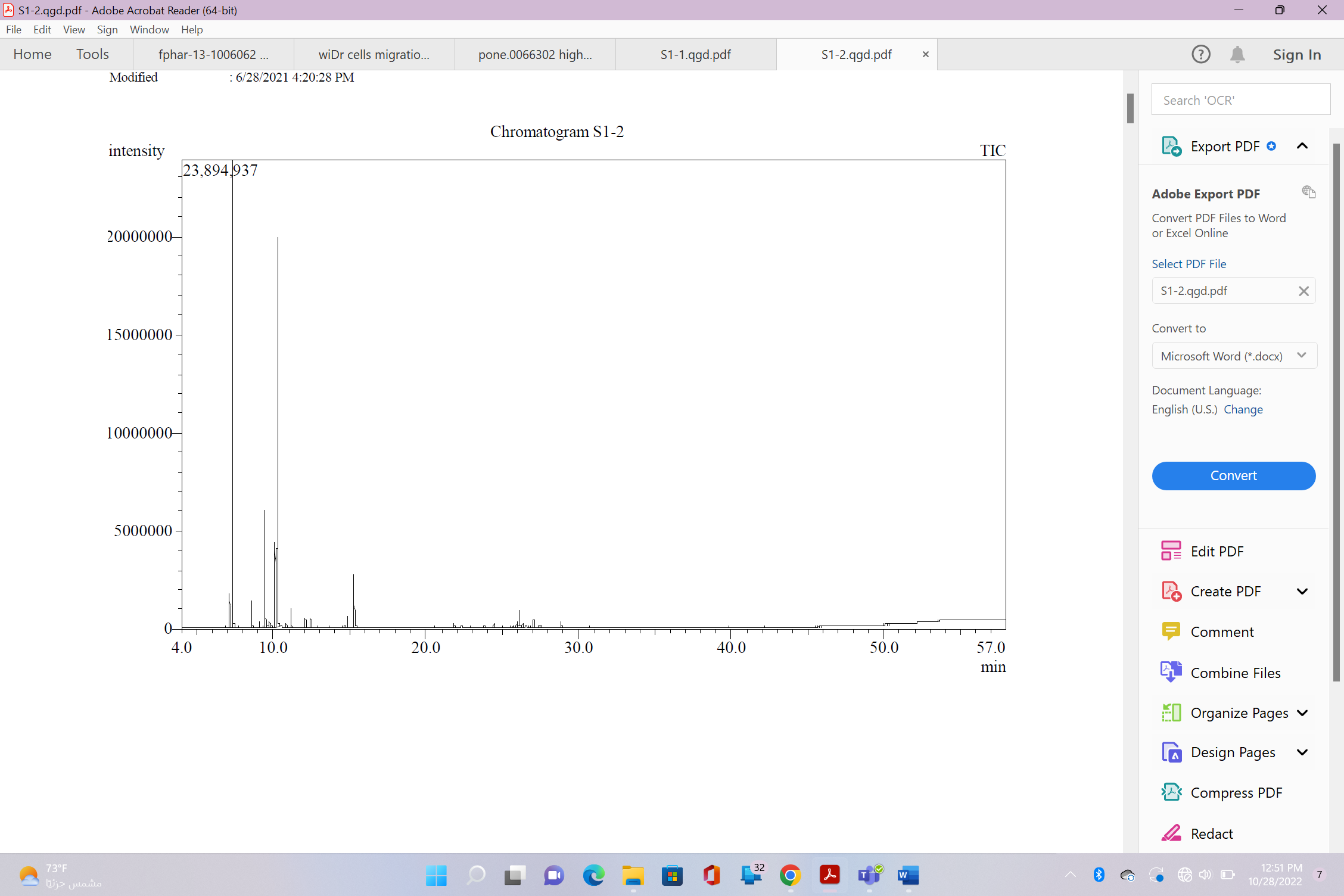
**

**Figure S1 B: Total ion chromatogram for *M. subulata* essential oil obtained by hydrodistillation (HD) extraction**

**
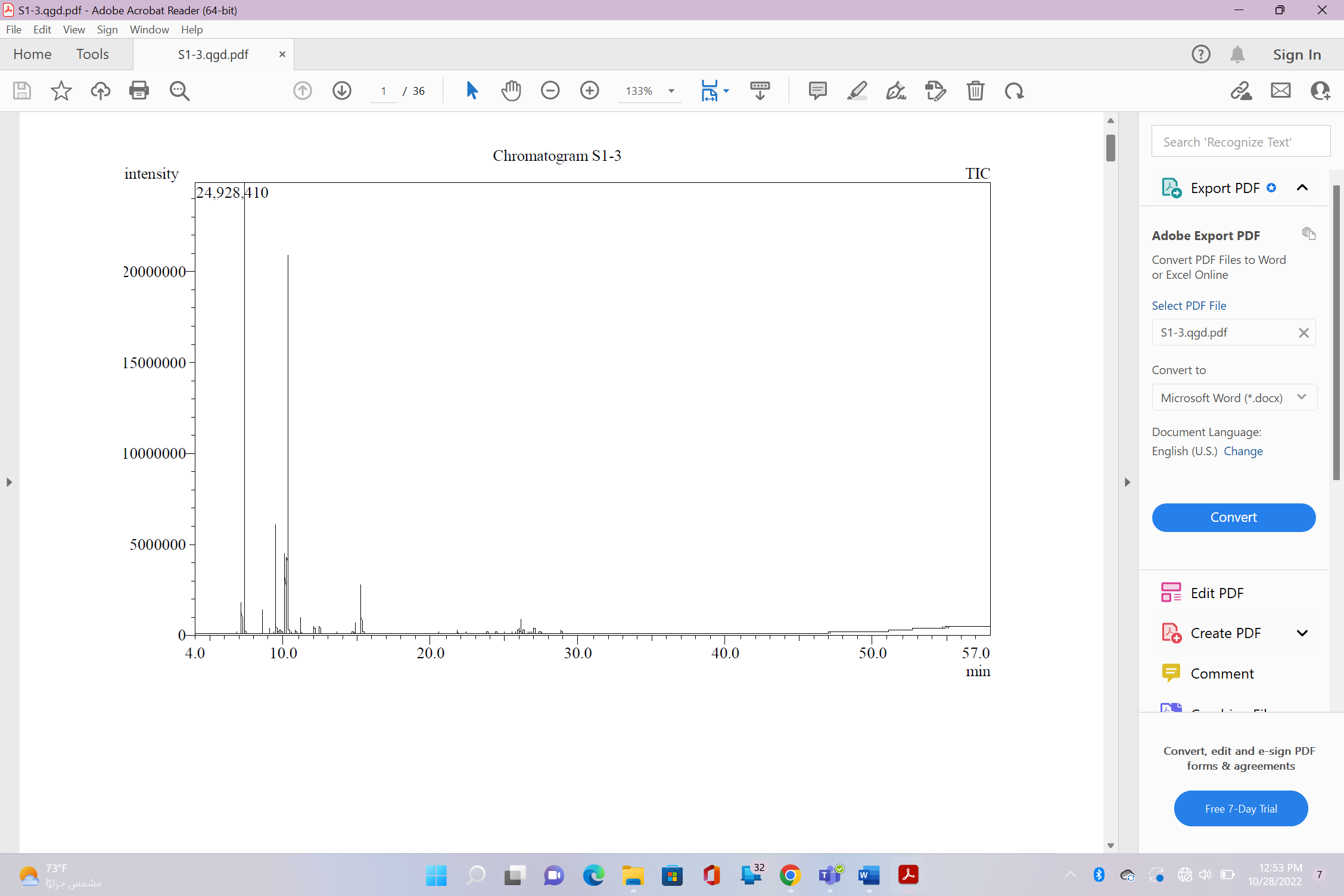
**

**Figure S1 C: Total ion chromatogram for *M. subulata* essential oil obtained by hydrodistillation (HD) extraction**

**
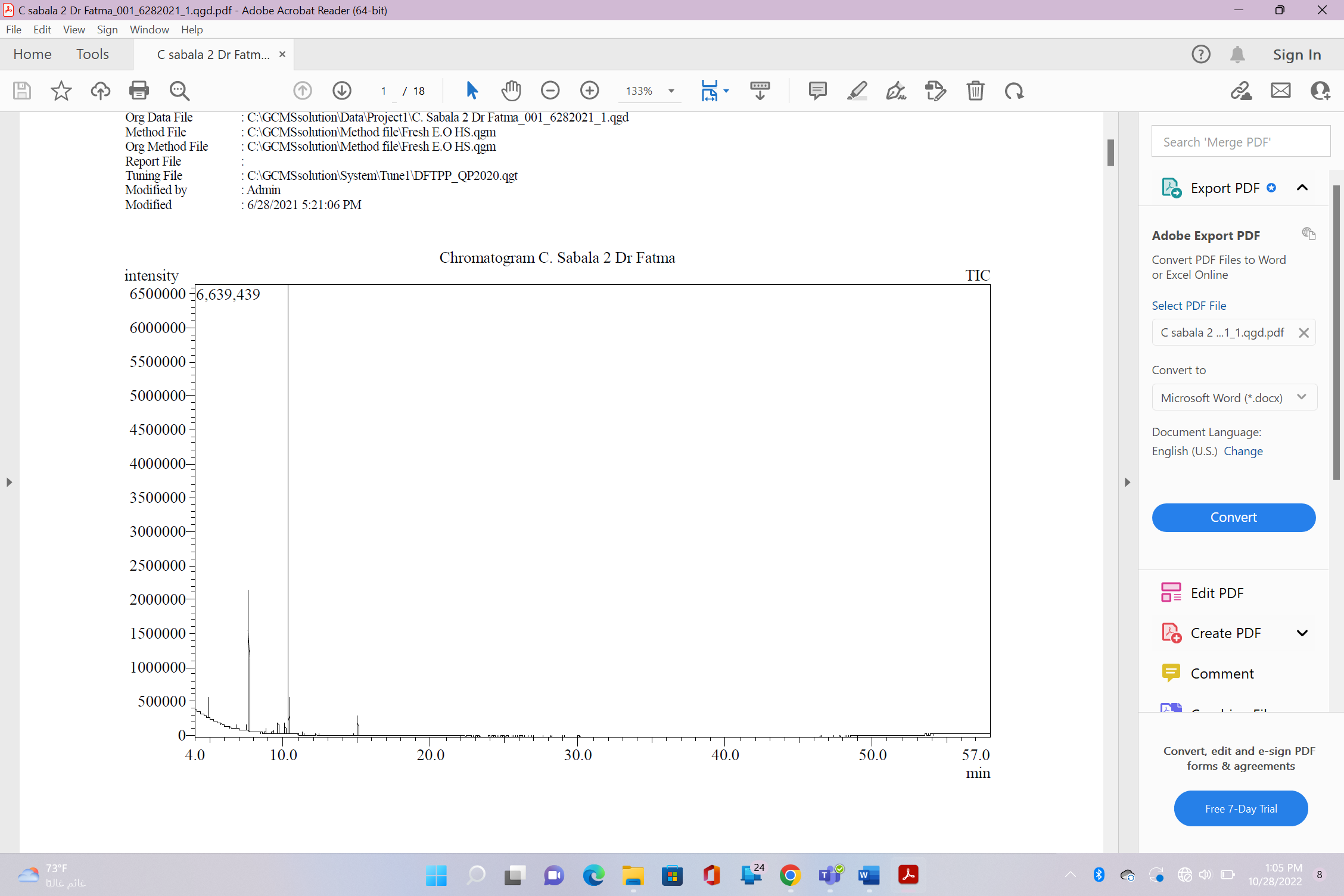
**

**Figure S2 A: Total ion chromatogram for *M. subulata* volatile constituents obtained by dynamic headspace (HS) extraction**

**
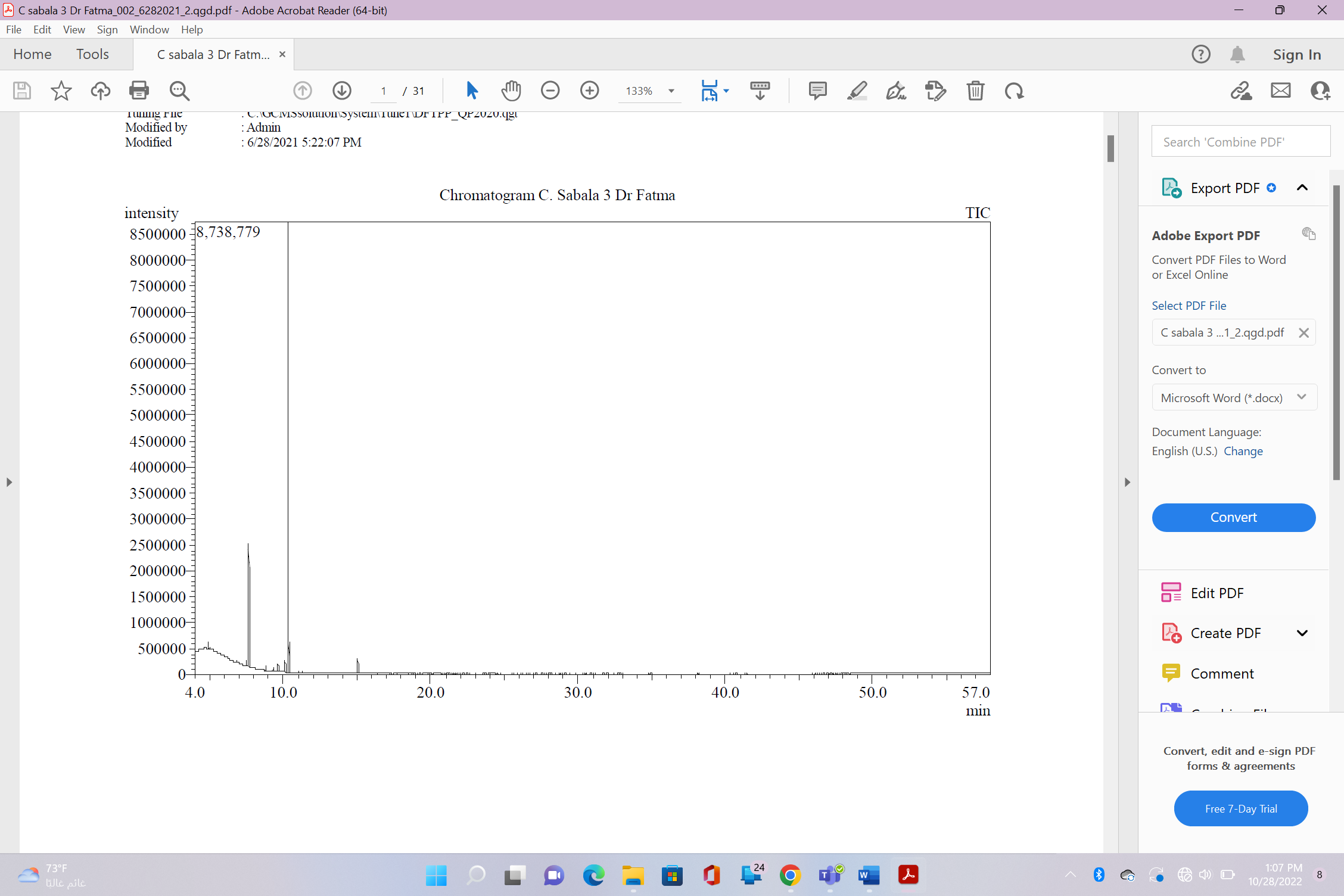
**

**Figure S2 B: Total ion chromatogram for *M.subulata* volatile constituents obtained by dynamic headspace (HS) extraction**

**
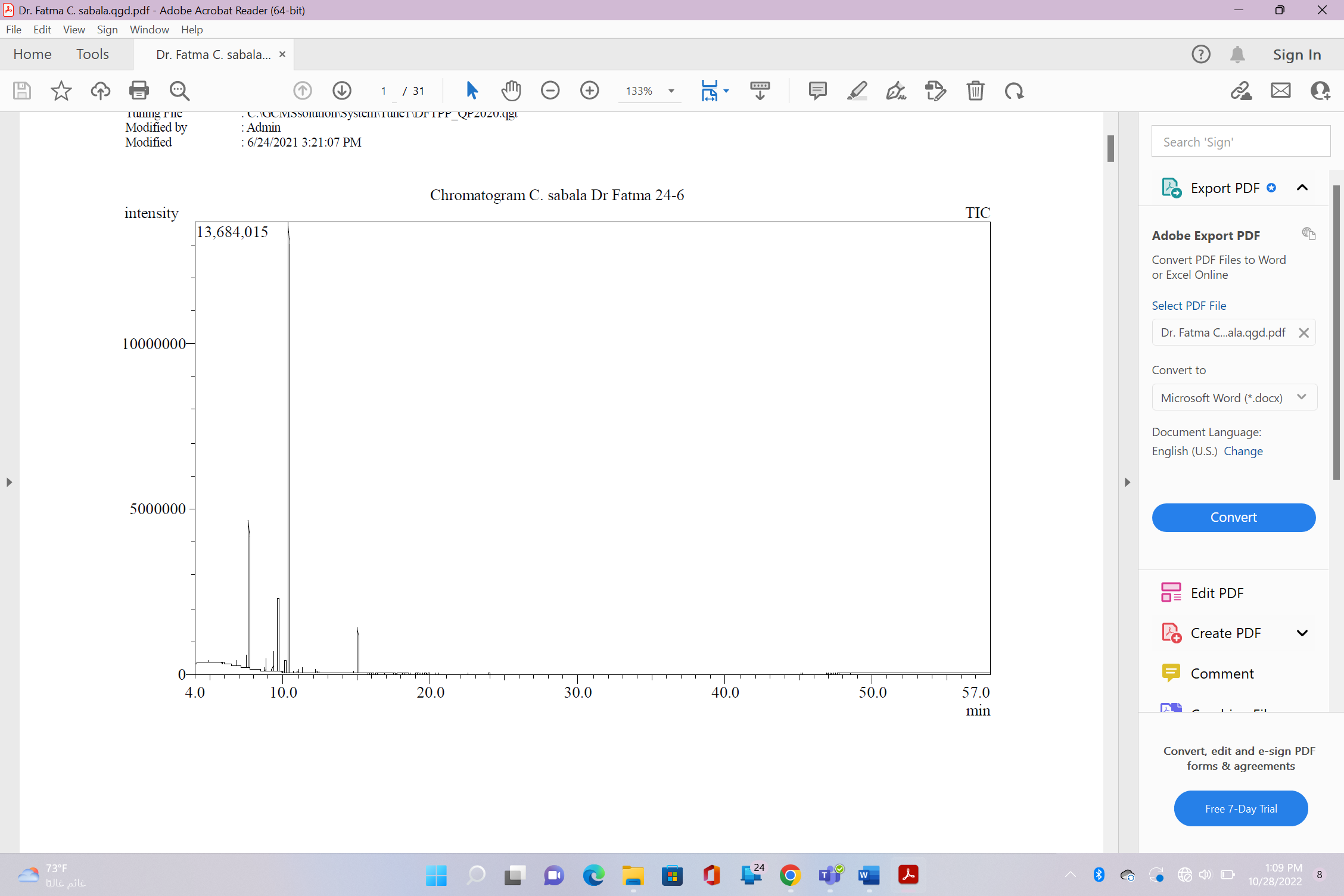
**

**Figure S2 C: Total ion chromatogram for *M.subulata* volatile constituents obtained by dynamic headspace (HS) extraction**


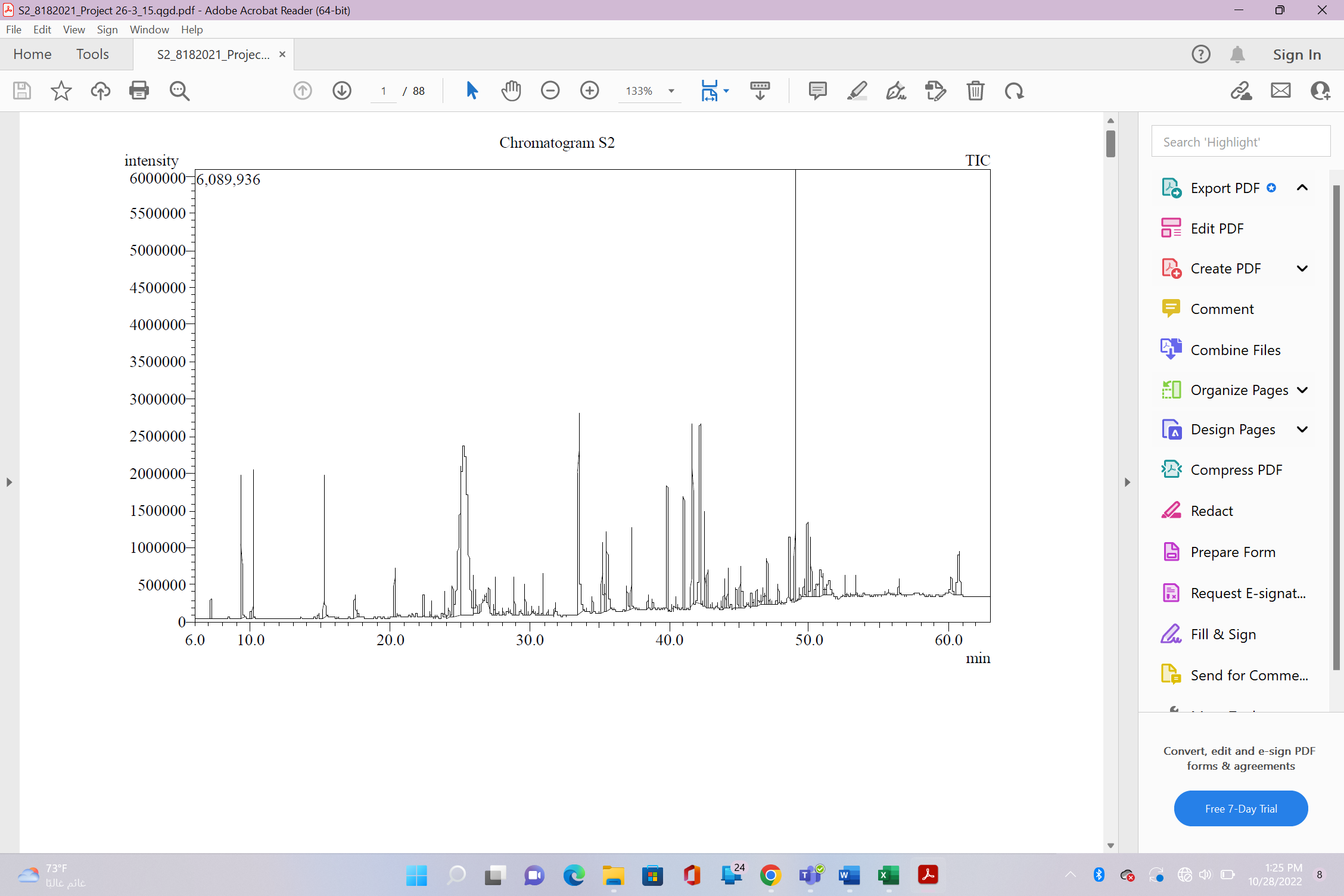


**Figure S3 A: Total ion chromatogram for *M.subulata* essential oil obtained by supercritical fluid (SF) extraction**


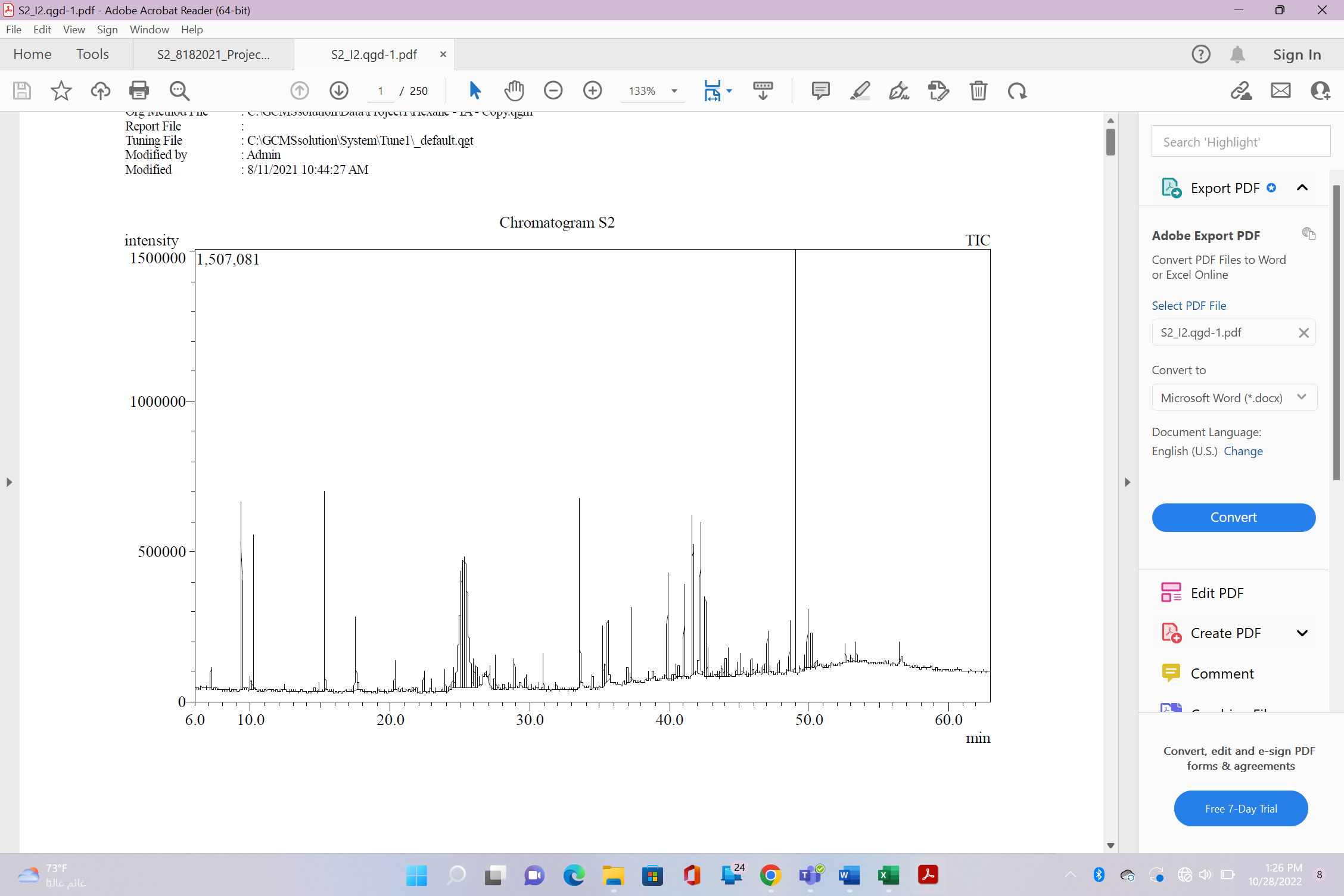


**Figure S3 B: Total ion chromatogram for *M.subulata* essential oil obtained by supercritical fluid (SF) extraction**


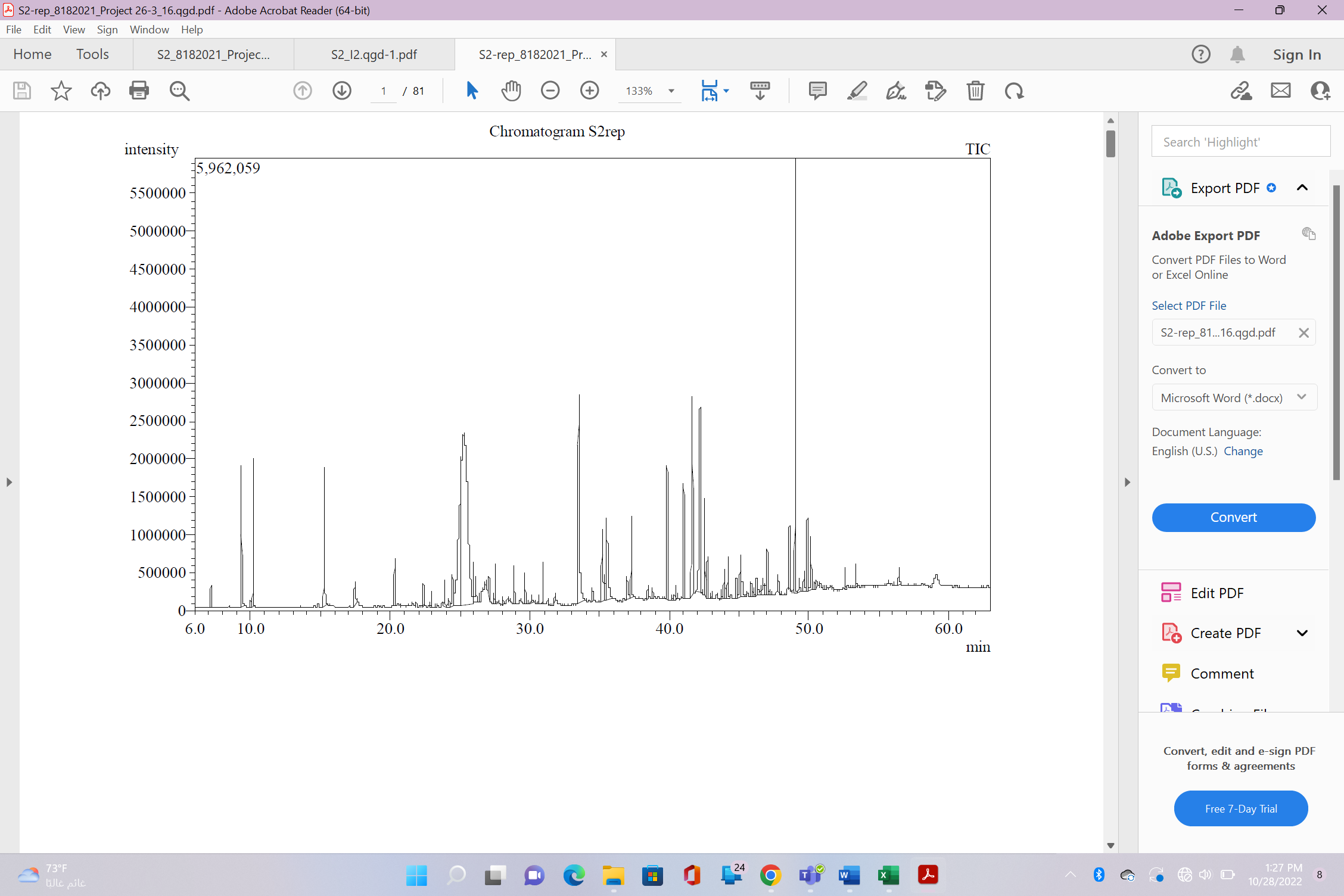


**Figure S3 C: Total ion chromatogram for *M.subulata* essential oil obtained by supercritical fluid (SF) extraction**

**Figure S4. Dose-response curve of the antimicrobial effect of *M.subulata* EOs obtained by conventional HD method in broth microdilution assay.**

**Figure S5. Dose-response curve of the antimicrobial effect of *M.subulata* EOs obtained by SF extraction method in broth microdilution assay.**

**Detailed conditions of GC/MS analysis for hydrodistillation-EO**

Gas chromatography-mass spectrometry (GC/MS) data of the hydrodistilled EO was recorded on a Shimadzu GC/MS-QP2010 (Kyoto, Japan) linked to a quadrupole mass spectrometer (Shimadzu Corporation, Kyoto, Japan). The separation of the volatile components was implemented by means of Rtx-5MS column (30 m × 0.25 mm i.d. × 0.25-μm film thickness, Restek, United States). Initial column temperature was kept at 45 °C for 2 min (isothermal) and programmed to 300 °C at a rate of 5 °C/min and kept constant at 300 °C for 5 min (isothermal). The injector temperature was 250 °C. The helium carrier gas flow rate was 1.41 ml/min. All the mass spectra were recorded applying the following conditions: (equipment current) filament emission current, 60 mA; ionization voltage, 70 eV; ion source, 200°C. Diluted samples (1% v/v) were injected with split mode (split ratio 1: 15).

**Detailed conditions of GC/MS analysis for supercritical fluid-EO**

Gas chromatography-mass spectrometry (GC/MS) data of the supercritical extracted EO was recorded on a Shimadzu GC/MS-QP2010 (Kyoto, Japan) linked to a quadrupole mass spectrometer (Shimadzu Corporation, Kyoto, Japan). The separation was implemented by means of Rtx-5MS column (30 m × 0.25 mm i.d. × 0.25-μm film thickness, Restek, United States). The initial column temperature was kept at 50 °C for 3min (isothermal) and programmed to 300 °C at a rate of 5 °C/min and kept constant at 300°C for 10 min (isothermal). The injector temperature was 280 °C. The helium carrier gas flow rate was 1.37 ml/min. All the mass spectra were recorded applying the following conditions: (equipment current) filament emission current, 60 mA; ionization voltage, 70 eV; ion source, 220°C. Diluted samples (1% v/v) were injected with split mode (split ratio 1: 15).

**Detailed conditions of dynamic head-space GC/MS analysis**

A Shimadzu headspace sampler HS-20 coupled to a Shimadzu GCMS-QP2020 gas chromatograph mass spectrometer (Kyoto, Japan) was adopted and equipped with Rtx-1MS column (30 m × 0.25 mm id. × 0.25 µm film thickness) (Restek, Bellefonte, PA, USA). The oven temperature was held at 80 °C, while the sample line and transfer line temperatures were held at 150 °C. Headspace sampling was carried out under the following conditions: the equilibration, pressurizing, and needle flush time were set at 8, 2, and 5 min, respectively. Besides, GC/MS headspace analysis was performed under the following conditions: column oven temperature was held for 2 min at 45 °C then increased to 300 °C at a rate of 5 °C /min and kept constant at 300 °C for 5 min. Helium was used as a carrier gas at a flow rate set at 1.41 mL/min. A split ratio of 1: 25 was employed. APCI pressure was set at 50 kPa, ion source temperature at 200 °C, and interface temperature at 280 °C.
